# Protective effect of dietary L-carnitine supplementation on follicular development in the ovary of ewes with subclinical pregnancy toxemia

**DOI:** 10.5194/aab-69-265-2026

**Published:** 2026-04-30

**Authors:** Ali Osman Turgut, Merve Pekince Özöner, Davut Koca, Ebru Karakaya Bilen, Fatma İşbilir, Özgür Özöner, Ali Ünver, Mehmet Eroğlu, Çiğdem Bolaç, Muhammed Hasan Şirin, İhsan İşbilir

**Affiliations:** 1 Siirt University, Faculty of Veterinary Medicine, Department of Animal Science, Siirt, Türkiye; 2 Siirt University, Faculty of Veterinary Medicine, Department of Histology, Siirt, Türkiye; 3 Yuzuncu Yil University, Faculty of Veterinary Medicine, Department of Obstetrics and Gynecology, Van, Türkiye; 4 Çukurova University, Faculty of Veterinary Medicine, Department of Obstetrics and Gynecology, Adana, Türkiye; 5 Siirt University, Faculty of Veterinary Medicine, Department of Anatomy, Siirt, Türkiye; 6 Siirt University, Faculty of Veterinary Medicine, Department of Pathology, Siirt, Türkiye; 7 Siirt University, Faculty of Veterinary Medicine, Department of Obstetrics and Gynecology, Siirt, Türkiye; 8 Siirt University, Faculty of Veterinary Medicine, Department of Pharmacology and Toxicology, Siirt, Türkiye; 9 Ankara University, Institute of Health Sciences, Department of Veterinary Pharmacology and Toxicology, Ankara, Türkiye

## Abstract

This study examined the effects of subclinical pregnancy toxemia (SPT) on follicular development and evaluated the protective potential of dietary L-carnitine supplementation in ewes. Eighteen crossbred Hamdani ewes underwent estrous synchronization, natural mating, and pregnancy confirmation via ultrasonography on day 45 post mating. The ewes were fed according to the National Research Council (NRC) (2007) guidelines until day 100 of gestation, after which they were assigned to one of three groups: SPT (G1, n=6 ), SPT+L-carnitine (G2, n=6 ), and control (G3, n=6 ). Blood β -hydroxybutyrate ( β HBA) levels were measured on days 100 and 138. Following slaughter, ovarian tissues were collected, processed, stained with Harris hematoxylin and eosin, and imaged for histometric analysis. Follicle types, oocyte diameters, and zona pellucida (ZP) thickness were recorded. Primordial follicle numbers did not differ significantly among groups ( p>0.05 ). Primary follicles tended to be higher in G1 compared to G2 and G3, but this was not statistically significant ( p<0.05 ). Secondary follicles differed significantly, with G2 exhibiting higher counts than G1 and G3 ( p<0.05 ), indicating enhanced secondary follicle development with L-carnitine supplementation. Antral follicles were low across all groups, with no significant differences ( p>0.05 ), consistent with limited preovulatory follicle formation during pregnancy. Atretic follicles were more numerous in G1 than in G2 and G3 ( p<0.05 ), suggesting increased follicular regression in ewes with subclinical pregnancy toxemia (SPT). Secondary follicle oocyte diameters were larger in G2 and G3 than in G1 ( p<0.05 ), while antral follicle oocyte diameters showed no significant differences ( p>0.05 ). ZP thickness tended to be higher in G3 for both secondary and antral follicles, although differences were not significant between groups ( p>0.05 ). Pairwise effect sizes (Cohen's d ) also indicated a large effect of L-carnitine on secondary follicle and antral follicle development, and oocyte diameter; and a negative effect of subclinical pregnancy toxemia on follicle development. These results highlight the importance of adequate nutrition and metabolic support during late gestation and suggest that L-carnitine supplementation may enhance follicular growth and reproductive performance in ewes with SPT. Further studies are needed to clarify the mechanisms by which L-carnitine protects ovarian functions and follicular development.

## 1 Introduction

Small ruminants, particularly sheep and goats, play a critical role in the livelihoods, economic stability, and food security of many people, especially in developing countries. Meat production and quality traits in small ruminants are essential for the sustainability of animal production (Kosgey and Okeyo, 2007). In small ruminants, pregnancy is a complex process that involves numerous physiological changes in pregnancy-related tissues (Kandil et al., 2025; İşbilir et al., 2024). Various metabolic diseases occur during late pregnancy due to the increasing demands of the developing fetus. In sheep, late-gestation pregnancy toxemia is defined as a nutrition-related metabolic disorder resulting from disruptions in carbohydrate and fatty acid metabolism (Rook, 2000; Ji et al., 2023). The disease often leads to fetal losses and, in some cases, maternal mortality, generating significant economic consequences (Moghaddam and Hassanpour, 2008; Abreu-Palermo et al., 2021). However, the subclinical form of the disease is much more prevalent under field conditions (Irmak et al., 2025). Blood β HBA levels are considered the gold standard for disease diagnosis (Turgut et al., 2024). Our previous findings indicated that exposure to SPT significantly reduces the number and diameter of fetal muscle fibers (Turgut et al., 2025a), potentially impairing postnatal growth and development (Turgut et al., 2025b). Additionally, PT in ewes is associated with maternal hypoglycemia (Iqbal et al., 2022) and increased triglyceride levels (Turgut et al., 2025b).

L-carnitine is a water-soluble, vitamin-like quaternary amine synthesized endogenously from lysine and methionine in the liver. It facilitates the transport of long-chain fatty acids across the mitochondrial membrane, supporting β oxidation and energy production, while also functioning as a co-factor and antioxidant, and acting as an important mitochondrial modulator (Pekala et al., 2011; Ringseis et al., 2018; Placidi et al., 2022). Previous studies have shown that L-carnitine supplementation improves biochemical and hematological parameters in pregnant ewes and enhances serum antioxidant capacity (Halawa et al., 2023). In another study, prepartum L-carnitine administration in sheep decreased serum NEFA concentrations without altering β HBA, triglyceride, or glucose levels (Pancarcı et al., 2007). These findings suggest that L-carnitine is a promising supplement for supporting energy metabolism, oxidative balance, and neonatal adaptation during pregnancy.

In mammals, follicular development continues during pregnancy, although it is markedly regulated by endocrine factors. Because the transformation from primordial to secondary follicles is primarily independent of gonadotropin stimulation, early stages of follicular development continue throughout gestation (Fortune, 1994; Gougeon, 1996). These follicles develop slowly and remain morphologically inactive within the ovarian cortex. Conversely, the later gonadotropin-dependent stages, particularly antral follicle growth and dominance, are inhibited by the elevated concentrations of progesterone and estrogens produced by the corpora lutea and placentae (Driancourt et al., 2000; Smith and Clarke, 2010). This hormonal environment suppresses the hypothalamic–pituitary axis, reducing the secretion of the gonadotropin-releasing hormone (GnRH), luteinizing hormone (LH), and follicle-stimulating hormone (FSH), thereby preventing ovulation. Studies in ruminant species such as sheep and cattle reveal that small antral follicles can still be found during pregnancy, but they generally become atretic instead of achieving preovulatory maturity. Therefore, while early follicular activity continues, the development of mature and ovulatory follicles is suppressed to maintain hormonal stability and the continuation of pregnancy (Driancourt et al., 2000; Bartlewski et al., 2011). However, the third trimester of gestation is the period when ewes require high energy and nutrients due to the rapidly developing fetus. However, weak pasture conditions may not meet high energy demands of ewes under extensive management (Irmak et al., 2025). Therefore, metabolic diseases during late pregnancy may negatively affect ongoing follicular development in sheep ovaries. SPT is a potential metabolic disorder that may impair follicular development during pregnancy. Furthermore, no comprehensive data exist on the effects of L-carnitine on follicular development in ewes with SPT during late pregnancy.

Experimental studies evaluating the effects of L-carnitine on follicular development are limited. Numerous in vitro studies in different species have demonstrated that L-carnitine supports oocyte maturation and embryonic development (Xu et al., 2018; Modak et al., 2022; Hao et al., 2025; Akter et al., 2025). This study aimed to investigate the effects of dietary L-carnitine supplementation on follicular development in late-gestation ewes affected by SPT in an experimental model.

## 2 Material and methods

### 2.1 Animals

The experiment was carried out at the Small and Large Ruminant Application and Research Center of Siirt University between September and October 2023. The research facility is located at $37{}^{\circ}58^{\prime }05^{\prime \prime }$ N, $41{}^{\circ}50^{\prime }14^{\prime \prime }$ E in Siirt, Türkiye. All procedures were performed during the autumn season, which corresponds to the natural breeding period for sheep. A total of 18 crossbred Hamdani ewes, aged between 2 and 3 years, and with initial body condition scores (BCSs) ranging from 2.75 to 3.25, were included in the study. The ewes were weighed and subjected to a nutritional flushing program starting 2 weeks prior to mating and continuing for 3 weeks post mating, in accordance with National Research Council (NRC) guidelines. All animals were clinically healthy and free from reproductive disorders. They were maintained in a well-ventilated housing facility, with unrestricted access to fresh water throughout the study period. Feeding was carried out twice daily: in the morning and evening.

### 2.2 Estrous synchronization and pregnancy diagnosis

To synchronize estrus, all ewes received intravaginal sponges containing 60 mg of medroxyprogesterone acetate (Esponjavet^®^, HIPRA, Türkiye), which remained in place for 6 d. After sponge removal, each ewe was injected intramuscularly with 500 IU of equine chorionic gonadotropin (eCG; Oviser^®^, HIPRA, Türkiye) and 50 µg of prostaglandin F2 α (PGF2 α ) analog, cloprostenol (Gestavet^®^, HIPRA, Türkiye). Estrus detection began 24 h post removal and was performed three times daily.

All ewes displayed signs of estrus and were bred naturally using two fertile rams. Those that mated were marked with ear tags and housed separately for efficient management. Pregnancy was diagnosed on day 45 following mating using transrectal ultrasonography. A real-time B-mode ultrasound scanner (Honda, HS-102V, Japan) with a 7.5 MHz linear transducer was used for pregnancy confirmation. We detected that of all the animals, 15 ewes carried a single fetus, while three ewes were carrying twin fetuses.

### 2.3 Groups

Until the 100th day of gestation, all ewes were fed based on the guidelines of the National Research Council (NRC, 2007). Thereafter, they were randomly divided into three groups: the subclinical pregnancy toxemia group (Group 1; G1, n=6 ), the treatment group receiving 5 g per head per day L-carnitine supplementation (Group 2; G2, n=6 ), and the control group (Group 3; G3, n=6 ). Ewes in G1, G2, and G3 were respectively fed 1.5 kg straw and 400 g barley; 1.5 kg straw, 400 g barley, and 5 g per head per day L-carnitine; and 1.2 kg straw, 400 g barley, and 400 g mixed feed. The nutritional content of the diet for each group is given in the Table 1. Due to random grouping, it was determined that the three animals carrying the twin fetuses were included in G3. Therefore, a total of nine fetuses were obtained from G3, whereas six fetuses were obtained from each of G1 and G2.

**Table 1:** Nutritional content of the ration in G1, G2, and G3.

	G1	G2	G3
Dry matter (DM) (kg)	1.45	1.45	1.54
Metabolizable energy (kcal)	2710	2710	3520
Crude protein (CP) (g)	75	75	135
Calcium (Ca) (g)	1.96	1.96	7.60
Phosphorus (P) (g)	1.90	1.90	4.18
Ca / P	1.03	1.03	1.62
ADF (%)	7	7	12
NDF (%)	20	20	22
L-carnitine (g)	–	5	–

### 2.4 β HBA measurement

Blood samples collected on the 100th and 138th days of the gestation. Collected samples centrifuged at 3000×g for 20 min. Then, serum was separated into sterile tubes and stored at -20 °C until further analysis. The diagnosis of subclinical pregnancy toxemia (SPT) in ewes was based on the β HBA concentrations determined by the reference laboratory test (Ranbut, Randox, UK). Prior to testing, the device was calibrated using the assay's standard calibrator. A β HBA level of 0.8 $\mathrm{mmol}\;L^{-1}$ was considered the critical threshold, and ewes with β HBA concentrations between 0.8 and 1.6 $\mathrm{mmol}\;L^{-1}$ were classified as having SPT (Xue et al., 2019). The intra-assay coefficient of variance (CV) of analysis was detected as 7 %.

### 2.5 Collection of tissue samples and histometric measurements

On day 138 of the study, the animals were slaughtered at a local slaughterhouse, and the ovarian tissue was promptly placed in 10 % formalin solution. Subsequently, the samples were processed by embedding in cassettes and following the standard tissue processing protocol. Afterward, tissues were embedded into paraffin blocks. Five separate sections with a thickness of 5 µm were obtained from each ovarian tissue, with at least 80 µm intervals between each section. The sections were stained using the Harris hematoxylin and eosin staining method. Follicles in the stained sections were counted according to the method described by Zhuang et al. (2010).

**Figure 1:**
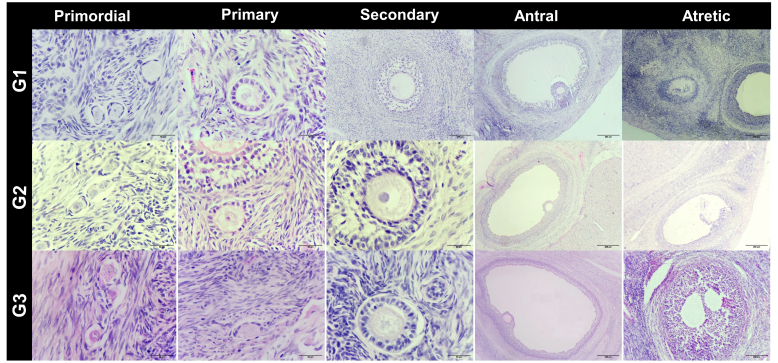
Histological images of ovarian tissue from G1: subclinical, G2: subclinical plus L-carnitine, and G3: control groups. Staining: H&E. Scale bars: 50 µm , 200 µm .

**Figure 2:**
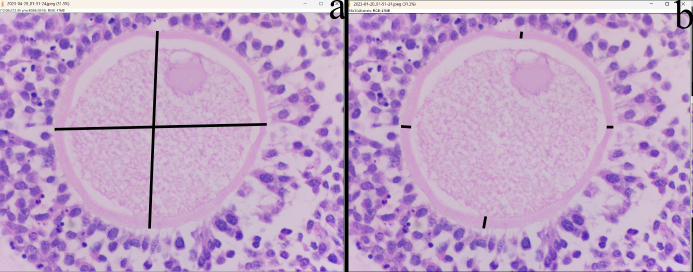
Histological image of ovarian histological sections showing oocyte diameter measurement **(a)** and zona pellucida thickness **(b)**. (H&E) bar: 40× .

In the cortical layer of the ovarian sections, follicles in which the oocyte nucleus and nucleolus could be distinctly identified were classified as primordial, primary, secondary, antral, or atretic. Structures surrounded by a single layer of squamous epithelial cells were identified as primordial follicles, whereas those surrounded by single or multiple layers of cuboidal epithelial cells were classified as primary follicles. Follicles with a developing antrum filled with follicular fluid were identified as secondary follicles. Follicles with a large antrum, and clearly distinguishable corona radiata and cumulus oophorus cell clusters were classified as antral follicles. Structures showing a loss of structural integrity, degenerating granulosa cells, and pyknotic nuclei were identified as atretic follicles (Kandil et al., 2024) (Fig. 1). In the images obtained from the sections, the diameter of oocytes and the thickness of the zona pellucida at the antral follicle stage were measured according to Griffin et al. (2006) (Fig. 2).

### 2.6 Statistical analysis

Minitab was used for all statistical analysis. Data distribution was evaluated with Shapiro–Wilk normality test. One-way analysis of variance (ANOVA) was used to compare normally distributed data, while a Kruskal–Wallis test was used to compare non-normally distributed data between groups. In a comparison of primordial, primary, secondary, atretic follicle number, oocyte diameter, and zona pellucida thickness, one-way ANOVA was performed. A Kruskal–Wallis test was performed and used to compare the antral follicle number between groups. A paired t test was performed to compare serum β HBA levels between the 100th and 138th days. The statistical significance level was set up as p<0.05 . GPower (v3.1) was used to calculate the effect sizes of the tests. Effect size f was calculated ANOVA, while Cohen's d was calculated pairwise comparisons. Effect size f classification was accepted as follows: 0.10–0.24: small effect, 0.25–0.39: medium effect, and >0.40 : large affect. Meanwhile, effect size $\eta^{2}$ classification was accepted as follows: $\eta^{2}=0.01$ : small effect, $\eta^{2}=0.06$ : medium effect, and $\eta^{2}=0.14$ : large effect. Cohen's d classification was accepted as 0.20–0.50: small effect, 0.51–0.79: medium effect, and >0.80 : large effect.

## 3 Results

There were no significant differences on serum β HBA between groups at the 100th day of pregnancy. At the 138th day, the serum β HBA level was greater than 0.8 $\mathrm{mmol}\;L^{-1}$ (1.01 ± 0.02 $\mathrm{mmol}\;L^{-1}$ ), confirming the SPT model in G1. However, the β HBA level was lower than 0.8 $\mathrm{mmol}\;L^{-1}$ in G2 and G3.

Follicle populations were assessed across the three experimental groups (G1, G2, and G3). Primordial follicles were abundant in all groups, with no significant differences observed ( p>0.05 ), indicating that the basic follicle reserve was maintained. Primary follicles tended to be higher in G1 compared to G2 and G3, although this difference did not reach statistical significance ( p>0.05 ), suggesting a biologically relevant trend. Secondary follicles differed significantly among groups, with G2 showing markedly higher counts than G1 and G3 ( p<0.01 ), indicating enhanced secondary follicle development in G2. Antral follicles were relatively low in all groups, with no significant differences ( p>0.05 ), consistent with limited preovulatory follicle formation during pregnancy. Atretic follicles were significantly more numerous in G1 compared to G2 and G3 ( p<0.05 ), suggesting increased follicular regression in this group (Table 2).

**Table 2:** Comparison of follicle numbers between groups.

Follicle type	G1	G2	G3	p value	Effect size ( $f/\eta^{2}$ )
Primordial	$102.83\pm 8.96^{a}$	$96.83\pm 9.24^{a}$	$104.3\pm 14.0^{a}$	0.87	0.12 ( f , small effect)
Primary	$8.00\pm 0.68^{a}$	$3.83\pm 1.40^{a}$	$4.83\pm 1.45^{a}$	0.07	0.59 ( f , large effect)
Secondary	$1.83\pm 0.48^{b}$	$6.83\pm 1.42^{a}$	$1.83\pm 0.70^{b}$	0.002^∗∗^	1.00 ( f , large effect)
Antral	$1.17\pm 0.40^{a}$	$1.83\pm 0.70^{a}$	$0.50\pm 0.22^{a}$	0.29	0.02 ( $\eta^{2}$ , small effect)
Atretic	$12.00\pm 1.32^{a}$	$7.50\pm 1.88^{\text{ab}}$	$5.33\pm 1.28^{b}$	0.021^∗^	0.74 ( f , large effect)

^∗^ p<0.05 . ^∗∗^ p<0.001 . f : effect size. $\eta^{2}$ : eta squared. ^a^, ^b^ different superscripts in rows indicate significant differences.

Regarding follicle size, secondary follicles had a significantly larger diameter in G2 and G3 compared to G1 ( p<0.05 ), whereas antral follicle oocyte diameters did not differ significantly among groups ( p>0.05 ). In addition, Cohen's d between G1–G2 and G1–G3 were 0.97 and 0.90 for secondary follicle oocyte diameter, and 0.89 and 0.95 for antral follicle oocyte diameter, respectively. Zona pellucida thickness tended to be higher in G3 for both secondary and antral follicle oocytes compared to G1 and G2, although differences were not statistically significant ( p>0.05 ) (Table 2). In pairwise comparison, effect sizes (Cohen's d ) between G1–G2 and G2–G3 for secondary follicle oocyte diameter were 0.97 and 0.90, respectively. For antral follicle oocyte, diameter effect sizes for G1–G2 and G2–G3 were calculated as 0.89 and 0.33, respectively. Effect sizes ( f and $\eta^{2}$ ) are summarized in Tables 2 and 3.

**Table 3:** Comparison of diameter follicles and thickness of ZP between groups.

	G1	G2	G3	p value	Effect size f
Diameter of secondary follicle oocyte ( µm )	$89.20\pm 12.2^{b}$	$118.16\pm 5.07^{a}$	$115.93\pm 8.50^{\text{ab}}$	0.035^∗^	0.50 (large effect)
Diameter of antral follicle oocyte ( µm )	$156.50\pm 11.0^{a}$	$180.74\pm 6.91^{a}$	$163.50\pm 3.14^{a}$	0.128	0.41 (large effect)
Thickness of ZP in secondary follicles ( µm )	$4.88\pm 0.29^{a}$	$5.64\pm 0.40^{a}$	$6.53\pm 0.88^{a}$	0.230	0.40 (large effect)
Thickness of ZP in antral follicles ( µm )	$7.95\pm 0.37^{a}$	$7.93\pm 0.65^{a}$	$10.49\pm 1.93^{a}$	0.164	0.59 (large effect)

^∗^ p<0.05 . f : effect size. ^a^, ^b^ different superscripts in rows indicate significant differences.

## 4 Discussion

SPT is a common metabolic disorder in the sheep industry. Due to its subclinical nature, the disease is underestimated in the veterinary field. The effect of SPT on biological and physiological development processes is still unclear. In addition, only a few studies have investigated the outcomes of SPT in sheep. In previous studies, we detected that SPT may have a negative impact on fetal skeletal muscle development during the prenatal period (Turgut et al., 2025a). In addition, SPT impairs postnatal growth, development, and the survival of lambs during the postnatal period (Turgut et al., 2025b). Although the disease is more common in prolific sheep breeds, we revealed that the disease may occur in non-prolific sheep breeds due to extensive management (Irmak et al., 2025). Despite its high prevalence, the disease is often underestimated by breeders in the field. This highlights the importance of protective agents to compensate for the negative effects of SPT in sheep. In a previous study, we detected that L-carnitine may support the fetal skeletal muscle development of fetuses during the prenatal period. L-carnitine also regulates the energy metabolism of pregnant ewes (Turgut et al., 2025a, b). However, data on the effect of SPT and L-carnitine are quite limited.

Nutrition is a fundamental physiological determinant in regulating follicular development in farm animals, and the levels of energy, protein, and micronutrients play a critical role in altering follicle diameter. Studies conducted in cattle clearly demonstrate that a negative energy balance suppresses dominant follicle growth. Lucy et al. (1991) reported that dairy cows experiencing an energy deficiency exhibit reduced IGF-1 and LH concentrations, which results in smaller dominant follicles and delayed ovulation. Similarly, Beam and Butler (1998) showed that elevated blood urea nitrogen caused by excessive protein intake adversely affects the biochemical composition of follicular fluid, leading to a reduction in follicle diameter and a decline in oocyte quality. The influence of nutrition on follicular response has long been investigated in sheep. Scaramuzzi et al. (2006) reported that the high-energy feeding strategy known as “flushing”, applied prior to mating, increases the number of large follicles and markedly stimulates ovarian activity. Energy restriction has been shown to cause follicles to remain small, whereas re-establishing an adequate energy level triggers a rapid follicular growth wave in cattle (Domingues et al., 2020).

In the Northern Hemisphere, the late gestation coincides with the winter season, when pasture is insufficient. During this period, breeders provide supplementary feeding to the sheep. However, for various reasons, animals are not provided with an adequate amount of feed. This situation may lead to SPT even in non-prolific sheep (Irmak et al., 2025). This suggests that the ongoing follicular development during late pregnancy may be affected due to subclinical pregnancy toxemia. In this experimental study, we detected that there were no significant differences in primordial and primer follicle numbers between groups. However, the secondary follicle number was significantly higher in the L-carnitine-supplemented group (G2) compared to G1 and G3. Meanwhile, the atretic follicle number was higher in the SPT group (G1) compared to G2 and G3. In addition, the secondary follicle and antral follicle oocyte diameter was greater in the L-carnitine-supplemented group (G2) compared to the SPT group (G1). Differences between G2 and G3 was not significant. These findings indicate that SPT may have a negative impact on the follicular reserve, and L-carnitine supports follicular development in ewes with SPT. In vitro studies support these findings. In buffalos, treatment with L-carnitine during in vitro oocyte maturation supports oocyte quality and embryonic development (Xu et al., 2018; Modak et al., 2022). In a study on mice, it was observed that injected L-carnitine supported maturation, fertilization rates, and the blastocyst cell number. Furthermore, novel in vitro studies revealed that L-carnitine improved maturation rates, fertilization rates of cattle, and goat oocytes collected from females with poor reproductive performance (Akter et al., 2025). Similarly, it was reported that L-carnitine supports the in vitro maturation of sheep (Bhakty et al., 2021; Hao et al., 2025) and camel oocytes (Fathi and Shahat, 2017). The findings from different in vitro studies support our findings and highlight the effects of L-carnitine. Catandi et al. (2023) reported that L-carnitine enhanced the developmental potential of bovine oocytes matured in vitro under high-lipid conditions. Reader et al. (2015) demonstrated that embryos derived from sheep oocytes matured in vitro with acetyl-L-carnitine (ALC) exhibited significantly higher cleavage, morula, and blastocyst rates during the post-fertilization period compared with zygotes cultured with L-carnitine (LC). Miyamoto et al. (2010) showed that oral L-carnitine (5 $\mathrm{mg}\;{\mathrm{mL}}^{-1}$ ) increased the number of ovulated oocytes, improved mitochondrial mass/distribution competence, and reduced oxidative damage in both oocytes and ovaries in mice with repeated ovulation cycles. However, to our knowledge, there are limited in vivo studies that evaluate the effect of L-carnitine supplementation on oocyte development in sheep. Essa et al. (2024) reported that L-carnitine may support follicle development and fertility in Barki sheep. To our knowledge, there is no obvious study investigating the effect of SPT and L-carnitine on follicle development in sheep. Therefore, the findings of this in vivo study may be valuable in showing the effects of metabolic diseases and L-carnitine on follicular development in sheep.

However, the underlying mechanism of the protective effect of L-carnitine on oocyte development is still unclear. Studies indicate that L-carnitine reduces oxidative stress in buffalo oocytes in vitro (Akter et al., 2025). Similarly, Hao et al. (2025) reported that L-carnitine upregulated genes associated with cellular responses to oxidative stress and DNA repair mechanisms in sheep. Reduced oxidative stress and increased in vitro oocyte maturation was also observed in women (Leitão et al., 2024). Furthermore, Mansour et al. (2008) reported that L-carnitine prevents DNA damage to oocytes incubated in the peritoneal fluid of endometriosis in women. Therefore, the protective effect of L-carnitine on follicular development in this study may be related to a similar mechanism in sheep and other species. However, this should be investigated in more comprehensive studies.

The zona pellucida (ZP) is a glycoprotein matrix that surrounds mammalian oocytes and plays a fundamental role in regulating the fertilization process. The thickness and structural integrity of the ZP are considered important indicators of oocyte quality, particularly in farm animals, and directly influence fertilization success. In cattle, decreasing oocyte quality has been shown to cause thinning of the ZP, which increases the risk of polyspermy (Santos et al., 2008). However, in sheep, reducing ZP thickness had no significant effect on the hatching of the embryos (Mousavi et al., 2022). In a previous study, ZP thickness of developing follicles was reported as 9.7 µm in sheep (Maside et al., 2021). In this study, we detected that ZP thickness of 10.49 and 6.53 µm in antral and secondary follicles of G3, respectively. However, in G1, ZP thickness was tented to be lower compared to the control group ( f=0.40 and f=0.59 ). This indicates that lower quality oocytes in G1 may have a lower ZP thickness. Therefore, these findings may suggest potential implications for oocyte competence, which warrants further investigation. Conversely, studies conducted in cattle have demonstrated that an increase in ZP thickness significantly impedes sperm penetration. Parrish et al. (1986) reported that a thicker ZP prolongs the time required for spermatozoa to traverse the zona and leads to reduced fertilization rates. In addition, embryo transfer studies have found that blastocysts with a thick ZP exhibit reduced hatching (Hoelker et al., 2006). Research in goats and cattle indicates that the structural characteristics of the ZP are highly sensitive to environmental stressors (Akter et al., 2025). Similarly, among the oocyte/zygote morphometric parameters, ZP thickness effected blastocyst formation, and both excessively thin and excessively thick ZP have been shown to exert different negative effects in sheep (Maside et al., 2021). These findings may suggest potential implications of L-carnitine for oocyte competence, which warrant further investigation.

## 5 Conclusion

In summary, SPT negatively affects follicular development, oocyte quality, and zona pellucida (ZP) structure in ewes, potentially impairing reproductive outcomes. Our results show that SPT reduces the number and diameter of secondary follicles while increasing atretic follicles, underscoring the adverse effects of metabolic stress on follicular development. Notably, L-carnitine supplementation had a protective effect on promoting secondary follicle growth, supporting overall follicular development and possibly reducing oxidative stress-related damage. These findings emphasize the importance of proper nutritional and metabolic management during late gestation and suggest that L-carnitine supplementation could be an effective strategy in improving follicular health and reproductive performance in ewes with SPT, including pregnancy rate, lambing rate, and litter size. Further studies are needed to clarify the precise mechanisms by which L-carnitine exerts its protective effects on sheep ovaries and follicular development.

## Data Availability

The data sets generated for this study are available on reasonable request from the corresponding author.

## Code availability

The statistical code used in this study is available from the corresponding author upon reasonable request.
